# Post encephalitic parkinsonism following dengue viral infection

**DOI:** 10.1186/s13104-017-2954-5

**Published:** 2017-11-29

**Authors:** B. V. K. M. Bopeththa, U. Ralapanawa

**Affiliations:** Teaching Hospital, Peradeniya, Kandy, 70550 Sri Lanka

**Keywords:** Dengue haemorrhagic fever, Viral encephalitis, Parkinsonism

## Abstract

**Background:**

Incidence of dengue fever as well as dengue hemorrhagic fever is increasing in Sri Lanka especially among elderly population. As the number of cases is rising, rare complications of dengue illness also can be seen in clinical practice when compared to the past few years. Prompt identification and treatment of such complications is challenging due to lack of awareness and unavailability of standard treatment.

**Case presentation:**

69 years old man presented with acute onset fever and was diagnosed as having dengue illness. On the 4th day of illness, the disease was progressed into dengue haemorrhagic fever and recovered uneventfully. Although he recovered from primary illness, his general condition continued to deteriorate due to new onset of features of parkinsonism. Cerebrospinal fluid analysis and electro encephalogram showed evidence of encephalitis. Cerebrospinal fluid analysis also revealed positive IgM antibodies against dengue virus. Then the diagnosis of post encephalitic parkinsonism following dengue viral infection was made and started on. He was started on SINEMET (carbidopa 10 mg and levodopa 100 mg) half tablet 6 hourly started. After 1 week of treatment he showed marked improvement and then patient was discharged with further follow up plan.

**Conclusion:**

Although the management of dengue illness and dengue haemorrhagic fever is straightforward, recognition and treatment of rare complications like post encephalitic parkinsonism following dengue viral infection is difficult without great clinical suspicion.

## Background

Currently dengue illness is the most common public health concern in Sri Lanka. Up to now (from 01/01/2017 to 24/07/2017) 105,153 cases have been reported [1]. In history, the first serologically confirmed case has been reported in 1962 and first outbreak occurred in 1965. First major epidemic occurred in 1989 and became endemic since then. Cyclical epidemics occurred from 2002 onwards [2]. Actually dengue infection has multi system involvement. Because of multisystem involvement, dengue infection can have variety of atypical presentations including neurological manifestations. In this case report we present a case with post encephalitic parkinsonism following dengue infection.

## Case presentation

69 years old retired teacher presented to us with 3 days history of acute onset febrile illness with headache, myalgia, joint pain and loss of appetite. He is a known patient with Non Hodgkin lymphoma since 2013 and treatment has been completed. He was regularly followed up in oncology clinic at Teaching hospital Kandy, Sri Lanka. He is a non smoker as well as never used alcohol. He was not on medications in the recent past. He was well and active at home prior to this admission. On admission he was conscious, rational walked to the admission room in a normal way. Body temperature was 101 °F and mild dehydration was present. Peripheries were warm with good capillary refill. Pulse rate was 80 beats per min with blood pressure of 130/80 mmHg. Chest, abdomen and neurological examination were unremarkable. Full blood count on admission showed white cell count of 8.9/μL with 80% of neutrophils and 13% of lymphocytes. Hemoglobin level was 10.2 g/dL. His platelet count was 200 cells/μL. Serum sodium level was 133 mmol/L and potassium level was 4.3 mmol/L. Serum creatinine was 84 μmol/L. C reactive protein level was within normal range. Liver enzyme results revealed aspartate aminotransferase (AST) level of 55 U/L and alanine aminotransferase (ALT) level of 26 U/L. Serum albumin and non fasting cholesterol levels were within normal range. Immediate ultrasound examination abdomen revealed no free fluid in the abdomen or pelvis. Dengue NS 1 antigen (non structural protein 1) was negative on admission.

Because of ongoing outbreak with high clinical suspicion, diagnosis of uncomplicated dengue fever was made and management started with intravenous normal saline, strict input and output chart maintenance and 6 hourly packed cell volume (pcv) monitoring according to national Dengue management guidelines.

On the 2nd day of admission (4th day of illness) he complained of postural symptoms, vomiting and increasing tiredness. On examination apart from mild dehydration other vital parameters were stable. With the suspicion of onset of critical phase ultrasound abdomen was performed and revealed thin layer of free fluid in hepatorenal pouch. The patient was taken into the high dependency unit and management was continued according to national guidelines. Throughout the critical phase his vital parameters, hourly urine output and hourly packed cell volume measurement remained stable. There was no indication for blood or blood products transfusion during this critical period. Table 1 demonstrates the laboratory results according to the hours spent in the critical phase. In addition to following, non fasting serum cholesterol was low and corrected serum calcium was within the normal range. Arterial blood gas analysis was normal.

**Table 1:** Laboratory results according to the hours spent in the critical phase

	WBC	Neu (%)	Lym (%)	PCV	Hb (g/dL)	Platelets	AST	ALT
0 h	7.8	77	14	33	10.6	186	57	31
12 h	6.8	67	32	34	10.8	160	59	40
24 h	7.1	70	29	34	11	120	57	39
36 h	5.59	65	34	33	10.4	100	62	35
48 h	6.24	66	33	32	11	173	60	38

On the 5th day of admission, Dengue IgM antibodies became positive and IgG was negative. Temperature also came to the base line. Although the patient recovered from dengue hemorrhagic fever, continued to deteriorate his general condition. His speech became slow and there was no energy in his purposeful movements. It took minutes to hours to get up from the bed. His care giver noticed increased stiffness of his arms and limbs. On examination he had expressionless face with cog wheel rigidity. He was severely bradikinetic and there was characteristic stoop posture with broad base gait when walking. Detailed neurological examination revealed Glasgow Coma Scale of 15/15 and there were no cranial nerve abnormalities. Sensory system examination also was normal. He was severely bradikinesic when performing finger nose testing and heel knee shin test.

Further laboratory investigations were performed as follows. Urgent MRI (magnetic resonance imaging) brain was done and was completely normal. EEG (electro encephalogram) showed evidence of encephalitis. Cerebrospinal fluid (CSF) analysis showed white blood cells (WBC) of 45 cells/mm^3^ (lymphocytes 92%), CSF protein of 58 mg/dL, no red cells and normal sugar level. CSF gram stain and bacterial cultures were negative CSF cytology for malignant cells was negative. CSF was tested for dengue IgM antibodies and it became positive CSF for herpes viruses, Japanese encephalitis virus, polio virus, coxsackie viruses and enteroviruses were negative. Rest of basic investigations including HIV (human immune deficiency virus) antibodies, ESR (erythrocyte sedimentation rate), C reactive protein level, serum electrolytes and full blood count were revealed no abnormality. According to the clinical picture the diagnosis of post encephalitic parkinsonism following dengue viral infection was made. He was started on SINEMET (carbidopa 10 mg and levodopa 100 mg) half tablet 6 hourly started. After 1 week of treatment he showed marked improvement and then patient was discharged with further follow up plan. After 4 weeks of treatment he showed almost complete recovery of his clinical symptoms.

## Discussion and conclusion

The dengue is an arbo viral infection caused by four different serotypes (DEN 1, DEN 2, DEN 3, and DEN 4). The dengue virus belongs to the flavivirus group and it is a single stranded enveloped RNA virus. The infection is transmitted by Aedes mosquitoes.

The dengue infection can have a presentation varying from a mild viral fever to dengue hemorrhagic fever and shock. And also it is a multisystem disease with variety of atypical presentations. Neurological manifestations are one such. They include Meningoencephalitis, Acute disseminated encephalomyelitis, Transverse myelitis and Guillain–Barre Syndrome. Encephalitis, the most common neurological manifestation, thought to occur in up to 6.2% of patients with dengue haemorrhagic fever but is rare in uncomplicated dengue fever [3]. Post encephalitic parkinsonism following dengue infection is one such rare presentation and there is very few case reports have been reported in the literature.

The parkinson’s disease is named after the English doctor called James Parkinson who published the first detailed description in an essay on the Shaking Palsy in 1817 [4]. Although parkinson’s disease has multiple etiologies, idiopathic parkinson’s disease is the commonest. Viral etiology is a known fact. It has been shown that numerous viruses can enter nervous system, i.e. they are neurotropic, and induce a number of encephalopathies. One of the secondary consequences of these encephalopathies can be parkinsonism. One of highlighted and controversial cases of viral parkinsonism is that which followed the 1918 influenza outbreak. Other viruses known to induce parkinsonism are coxsackie, Japanese encephalitis, West Nile, HIV, varicella zoster, Herpes Simplex, Epstein Barr and cytomegalovirus [5]. But acute onset parkinsonism following dengue viral infection less commonly reported.

How viruses actually cause parkinsonism still we do not know. Although efforts have been made to detect viral particles, in the brain, or antibodies in the serum or CSF of patient with parkinson’s disease, they have been generally unsuccessful. But recent immunohistochemical work has revealed the presence of complement proteins and the interferon induced MxA in association with Lewy bodies and swollen neuronal process. So we propose a hypothesis that neurovirulent influenza A and other potent viruses may be responsible for the formation of Lewy bodies and later cell death of nigral neurons, to constitute a viral etiology for parkinson’s disease [6].

Although this patient’s during 2nd day of illness, NS-1 antigen was negative, latter part of illness dengue IgM became positive. The reason may be, because sensitivity of NS-1 ELISA kits was found to be varying depending on the serological status of the patient (primary versus secondary dengue infection), time of specimen collection and dengue serotype [7]. Immunochromatographic method of detecting IgM antibodies shows excellent sensitivity (100%) in diagnosis of both primary and secondary dengue infections [8].

In this patient EEG showed generalized slow waves reflecting evidence of encephalitis. There are no reported characteristic EEG abnormalities found in dengue encephalitis in the literature. Although this patient’s MRI was normal, there are several case reports on striking MRI findings in dengue encephalitis such as transient splenial hyper intensity appearing as dot sign [9] and bilateral symmetrical thalamic lesions [10]. MRI findings in dengue encephalitis are mostly non specific and findings can be seen in Japanese encephalitis and herpes encephalitis [11]. In a patient with parkinson’s disease initial MRI findings are subtle, but loss of normal swallow tail appearance of susceptibility signal pattern in the substantia nigra on axial imaging is perhaps most promising sign [12].

As mentioned according to latest immunohistochemical studies, there is a possibility that potent viruses may be responsible for the formation of Lewy bodies and later cell death in nigral region [6]. So routine anti Parkinson medications like carbidopa and levodopa combination may be effective as illustrated by this case study.

Post encephalitic parkinsonism following dengue infection is a very rare presentation and only very few case reports are available. Although viral etiology may be responsible for Lewy body formation in substantia nigra, further studies are needed to clarify this matter. And that will help us to develop definitive treatment modality for such patients.

## Abbreviations

SINEMET: carbidopa 10 mg and levodopa 100 mg
AST: aspartate aminotransferase
ALT: alanine aminotransferase
Dengue NS 1 Ag: Dengue non structural protein 1 antigen
PCV: packed cell volume
MRI: magnetic resonance imaging
EEG: electroencephalogram
CSF: cerebro spinal fluid
WBC: white blood cell
HIV: human immunodeficiency virus
ESR: erythrocyte sedimentation rate
ELISA: enzyme-linked immunosorbent assay
